# A Study on the Interaction of Rhodamine B with Methylthioadenosine Phosphorylase Protein Sourced from an Antarctic Soil Metagenomic Library

**DOI:** 10.1371/journal.pone.0055697

**Published:** 2013-01-31

**Authors:** Paulina Bartasun, Hubert Cieśliński, Anna Bujacz, Anna Wierzbicka-Woś, Józef Kur

**Affiliations:** 1 Department of Microbiology, Faculty of Chemistry, Gdańsk University of Technology, Gdańsk, Poland; 2 Institute of Technical Biochemistry, Lódź University of Technology, Lódź, Poland; 3 Department of Microbiology, Faculty of Biology, University of Szczecin, Szczecin, Poland; Consejo Superior de Investigaciones Cientificas, Spain

## Abstract

The presented study examines the phenomenon of the fluorescence under UV light excitation (312 nm) of *E. coli* cells expressing a novel metagenomic-derived putative methylthioadenosine phosphorylase gene, called *rsfp*, grown on LB agar supplemented with a fluorescent dye rhodamine B. For this purpose, an *rsfp* gene was cloned and expressed in an LMG194 *E. coli* strain using an arabinose promoter. The resulting RSFP protein was purified and its UV-VIS absorbance spectrum and emission spectrum were assayed. Simultaneously, the same spectroscopic studies were carried out for rhodamine B in the absence or presence of RSFP protein or native *E. coli* proteins, respectively. The results of the spectroscopic studies suggested that the fluorescence of *E. coli* cells expressing *rsfp* gene under UV illumination is due to the interaction of rhodamine B molecules with the RSFP protein. Finally, this interaction was proved by a crystallographic study and then by site-directed mutagenesis of *rsfp* gene sequence. The crystal structures of RSFP *apo* form (1.98 Å) and complex RSFP/RB (1.90 Å) show a trimer of RSFP molecules located on the crystallographic six fold screw axis. The RSFP complex with rhodamine B revealed the binding site for RB, in the pocket located on the interface between symmetry related monomers.

## Introduction

Rhodamine B (RB) is a xanthene dye commonly used in the textile industry [1], and it is also used as a stain in microbiology [2], [3], histology and pathology applications [4], [5]. Moreover, RB is classified as a toxic chemical compound; therefore, the usage of RB as a colorant in food and cosmetic products is prohibited in most countries of the world. However, the several serious incidents of an unauthorized using RB as colorant in food were reported in recent years [6].

In 1987, Kouker and Jaeger presented a new plate assay dedicated to the detection of lipase producing bacterial species and to quantify lipase activity in culture supernatants [7], based on a medium containing olive oil and the fluorescent dye rhodamine B. In this assay, during growth of bacterial colonies producing lipase orange fluorescent halos appear when the plates are illuminated by UV light at 350 nm. The sources of this fluorescence are complexes of rhodamine B with the free fatty acids released from the olive oil by lipases. To date, this plate assay is commonly used for detection of lipolytic bacterial strains [8] and for screening of recombinant bacterial colonies with lipolytic activity in genomic DNA or metagenomic DNA libraries [9], respectively. Previously, we also used this assay to screen the metagenomic DNA library for novel lipolytic enzymes from uncultured soil microorganisms [10]. Surprisingly for us, one of the clones without any lipase activity showed a high level of pink fluorescence [11]. Our detailed study of this unexpected discovery revealed that the presence of rhodamine B in the screening medium and a metagenomic-derived *rsfp* gene expression in *E. coli* cells are the key factors for the pink fluorescence occurring under screening conditions. At that stage of research we had not yet found the connection between the RB molecules, RSFP protein and the pink fluorescence of the recombinant colonies. The reason of the failure were problems encountered with purification of the recombinant RSFP protein, produced as inclusion bodies in the *E. coli* BL21(DE3)pLysS strain [11]. Therefore, in this study we focused on the production of soluble purified RSFP protein, and the analysis of its spectroscopic properties. Simultaneously, we examined the spectroscopic properties of the fluorescent dye RB, especially in the presence of purified RSFP protein or *E. coli* proteins, respectively. Besides the spectroscopic studies, we carried out crystallographic studies of native RSFP protein and RSFP protein crystal soaked with RB.

The spectrophotometric studies have revealed that the RSFP/RB complex formation could be responsible for the fluorescence of *E. coli* cells expressing *rsfp* gene, owing to the change in the spectroscopic properties of the dye molecule binding on the RSFP protein. Next, the crystal structure of the RSFP/RB complex allowed to identify the two RB binding sites located in the vicinity of the highly flexible D222–D236 loop. The site-directed mutagenesis of amino acids D222 and D224 at the one of the proposed binding sites resulted in the lack of the pink fluorescence of *E. coli* colonies expressing the exact variants of *rsfp* gene and growing on the Luria Bertani agar plates supplemented with RB.

## Materials and Methods

### Bacterial strains and plasmids

The plasmid pPINKuv [11] was used as a template for PCR amplifications of the *rsfp* gene. The plasmid pBADMycHisA (Invitrogen) was used for *rsfp* gene cloning and expression using an arabinose induced promoter in the cells of *E. coli* strain LMG194.

### Expression and purification of recombinant RSFP protein in *E. coli*

**Figure 1:**
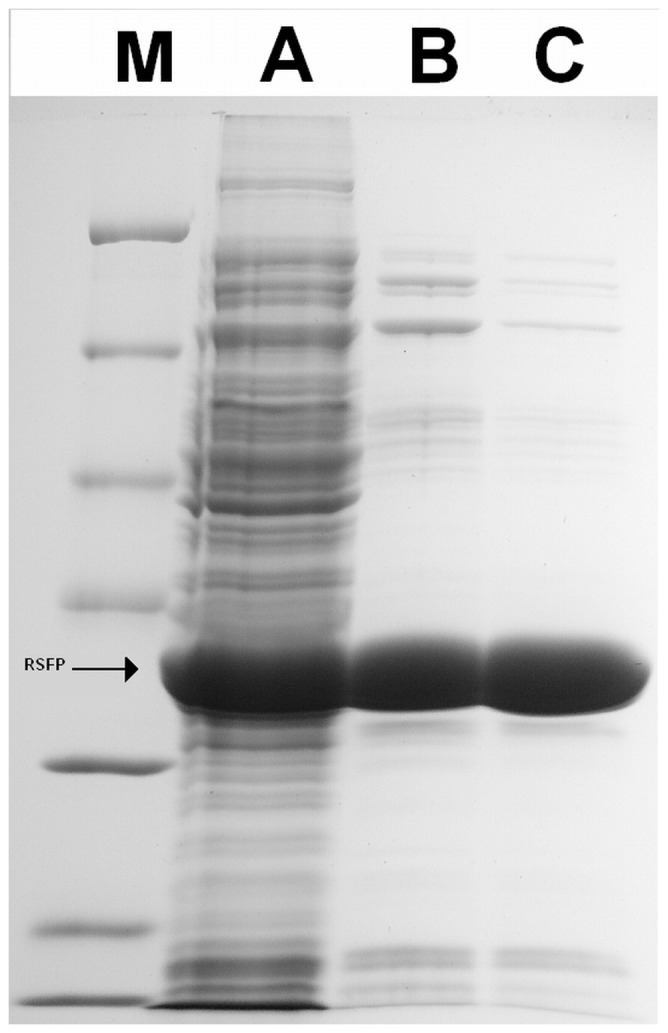
SDS-PAGE analysis of the cell extract with RSFP protein and the purified enzyme. Protein weight marker (lane M), cell extract of *E. coli* LMG194/pBADRSFP (lane A), pooled fraction after Fractogel EMD DEAE chromatography (lane B), pooled fraction after Resource Q chromatography (lane C).

The *rsfp* gene was amplified by PCR using primers: forward rsfp/pBAD (5′TTATCATGACTTCAACCGCAGTAGAAATTAC3′) and reverse rsfp/pBAD (5′ATACTGCAGCTACGGAAGTAATGCTGCAAGTCT3′). The amplified DNA fragments were digested with *Pag*I and *Pst*I enzymes, and then inserted into a pBADMycHisA vector. The resulting expression plasmid pBADRSFP was selected by way of restriction analysis. Next, the *rsfp* gene sequence was confirmed by DNA sequencing (Genomed, Poland). Afterwards, the pBADRSFP plasmid was used for production of the RSFP protein in the *E. coli* LMG194 strain. Cells were grown at 37°C in an LB medium (1% peptone K, 0.5% yeast extract, 1% NaCl) supplemented with tetracycline (12 µg mL^−1^) and ampicillin (100 µg mL^−1^). The *rsfp* gene expression was induced at OD_600_ = 0.5 with l-arabinose added to the final concentration of 0.2%, after which the culture cultivation was continued for 8 h at 30°C. Next, the culture was centrifuged (4000 r.p.m. for 15 min at 4°C) and the pellet was resuspended in a buffer A (0.02 M sodium phosphate buffer, 0.05 M NaCl, pH 6.3) and sonicated. The cell debris was collected by centrifugation at 10,000 r.p.m. for 20 min at 4°C and then the cell-free extract was applied to a Fractogel EMD DEAE column (Merck, Germany) previously equilibrated with an A buffer. An elution was carried out with a linear NaCl gradient (0.05–1.5 M) in the A buffer and with a flow rate of 1 mL min^−1^. The eluted fractions were monitored for the presence of the recombinant RSFP protein by SDS-PAGE. The fractions with the highest concentrations of protein corresponded to the expected molecular weight of RSFP protein were pooled, and then dialyzed against an A1 buffer (0.02 M sodium phosphate buffer, pH 6.3). Subsequently, the RSFP protein after the first step of purification was applied onto Resource Q column (Merck, Germany) previously equilibrated with buffer A. An elution was carried out with a linear NaCl gradient (0.1–0.6 M) in the buffer A, and with a flow rate of 0.5 mL min^−1^. Next, the eluted fractions containing the highest yield of target protein (analyzed by SDS-PAGE) were pooled and dialyzed to A1 buffer. Finally, the electrophoretic purity of the RSFP protein after the second step of purification was estimated by densitometry analysis of SDS-PAGE gels (Fig. 1) using ImageJ software, version 1.44l (NIH, USA).

### Estimation of molecular weight of RSFP protein

The purified enzyme was applied to a Superdex 200 10/300 GL gel-filtration column (Amersham Bioscience) pre-equilibrated with a 50 mM sodium phosphate buffer, 150 mM NaCl (pH 7.0). Gel filtration was conducted by high-performance liquid chromatography with the same buffer as the eluent with a flow rate of 0.5 mL min^−1^, and the elution patterns were compared with those of the standard proteins. As the standard proteins were used: thyroglobulin (*M*_r_ = 669,000 Da), apoferritin (*M*_r_ = 440,000 Da), β-amylase (*M*_r_ = 200,000 Da), alcohol dehydrogenase (*M*_r_ = 150,000 Da), bovine serum albumin (*M*_r_ = 66,000 Da), and carbonic anhydrase (*M*_r_ = 29,000 Da).

### The pink fluorescence assay

**Figure 2:**
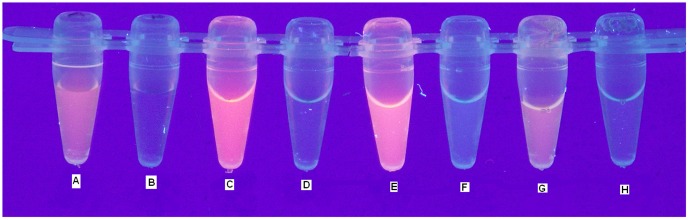
The pink fluorescence assay: RB in PBS buffer (A), PBS buffer (B), RB+RSFP in PBS buffer (C), RSFP in PBS buffer (D), RB+*E. coli* LMG194/pBADRSFP cell lysate in PBS buffer (E), *E. coli* LMG194/pBADRSFP cell lysate in PBS buffer (F), RB+*E. coli* LMG194/pBADMycHisA cell lysate in PBS buffer (G), and *E. coli* LMG194/pBADMycHisA cell lysate in PBS buffer (H).

The procedure presented below describes the final version of the pink fluorescence assay. The 93 µL of PBS buffer was mixed with 7 µL of RB solution in the PBS and named as a sample A. The 100 µL of the PBS buffer was used as negative control in this assay and named sample B. Next, the 73 µL of the purified RSFP protein, the *E. coli* LMG194/pBADRSFP cell lysate, and *E. coli* LMG194/pBADMycHisA cell lysate were mixed with a 7 µL of RB solution in the PBS buffer (5.0×10^−3^ g L^−1^) and 20 µL of PBS buffer and named samples C, E and G, respectively. Simultaneously, 73 µL of the purified RSFP protein, the *E. coli* LMG194/pBADRSFP cell lysate (the part of the same lysate used for purification of RSFP protein) and *E. coli* LMG194/pBADMycHisA cell lysate (used as a control for monitoring RSFP production in *E. coli* cells) were mixed with 27 µL of PBS buffer and named as samples D, F and H, respectively. Moreover, all samples and the control were vortexed and centrifuged (3000 r.p.m. for 30 s) and their fluorescence under UV light (312 nm) was captured by a Minolta Dimage A2 Super Fine (Fig. 2).

The final concentration of purified RSFP protein, the proteins of the *E. coli* LMG194/pBADRSFP cell lysate, and the *E. coli* LMG194/pBADMycHisA cell lysate in the corresponding samples was 2.3 g L^−1^, respectively. The final concentration of rhodamine B was also kept constant in all analyzed samples that contained this fluorescent dye. The rhodamine B (grade for fluorescence) used in this study was purchased from Sigma (USA).

### Absorption and emission spectra

**Figure 3:**
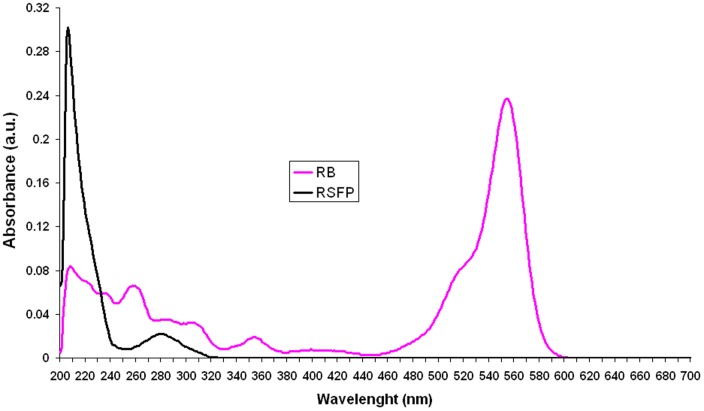
The UV-VIS absorption spectra of rhodamine B (RB; 1.75×10^−3^ g L^−1^) and RSFP protein (0.023 g L^−1^).

The UV-VIS absorption spectra of RB (1.75×10^−3^ g L^−1^, Fig. 3) and purified RSFP protein (0.023 g L^−1^, Fig. 3) in the range 200–700 nm were measured with an Evolution 300 UV-VIS spectrophotometer operated with double beam mode (Thermo Electron Corporation) using quartz cuvettes with 1.0 cm optical path length, respectively. In these measurements the reference cuvette contained PBS buffer. The emission spectra of RB (1.225×10^−3^ g L^−1^) and pure RSFP protein (0.081 g L^−1^) in the range of 400–750 nm (exc. 312 nm, bandwidth 9 nm) were measured with an Infinite 200 (Tecan Group Ltd.) spectrophotometer using 96-well microplates (BD Falcon™ Microplates black/clear, Cat. No. 353219) and following instructions in the manufacturer's manual. The temperature in all experiments was kept at 25°C.

The absorption and emission spectra of RB in the presence of RSFP protein or *E. coli* LMG194/pBADMycHisA proteins (the control sample) were obtained under conditions described above. In the all analyzed samples the concentration of the RB was also tightly controlled by mixing a constant volume of the RB stock solution (5.0×10^−3^ g L^−1^) with appropriate volumes of the RSFP stock solution (3.2 g L^−1^) or *E. coli* LMG194/pBADMycHisA proteins stock solution (2.3 g L^−1^), and filled up to total volume of 2 mL with PBS. However, due to the use of the double beam UV-VIS spectrophotometer, it is important to note that the reference cuvettes used to measure the absorbance of the background solutions for RB contained the same concentrations of *E. coli* proteins or RSFP protein, respectively, as in the cuvettes with the analyzed samples that additionally contained the same concentration of the analyzed dye.

**Figure 4:**
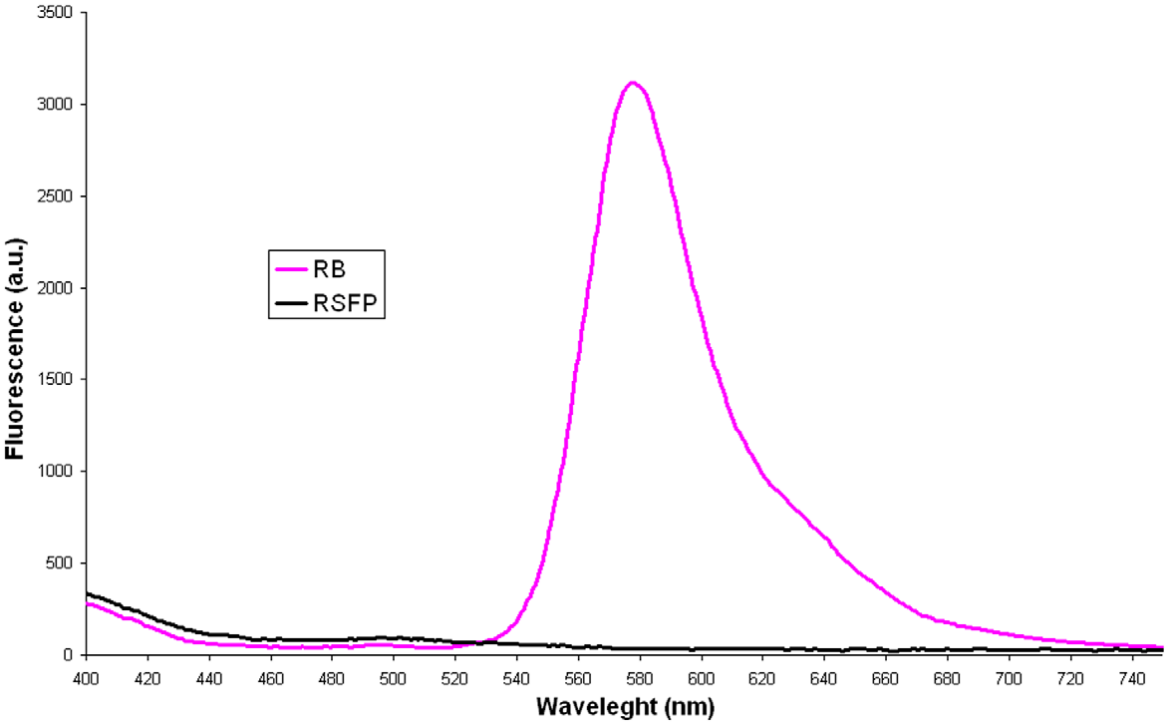
The emission spectra of rhodamine B (RB; 1.225×10^−3^ g L^−1^) and RSFP protein (0.081 g L^−1^).

**Figure 5:**
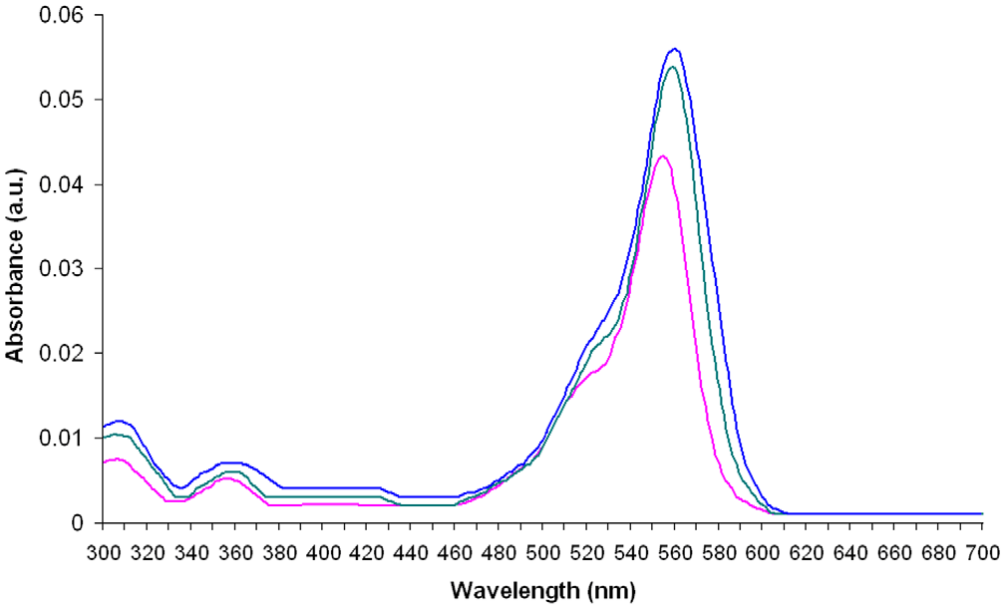
The UV-VIS absorption spectra of rhodamine B (green and blue lines) in the presence of the different concentration of RSFP protein, respectively, and the UV-VIS absorption spectrum of rhodamine B in the absence of RSFP protein (pink line). The concentration of RSFP protein in the first analyzed sample (green line) was twice higher than the concentration of RSFP protein (0.023 g L^−1^) in the second one (blue line), respectively. The concentration of RB (3.5×10^−4^ g L^−1^) was the same in the all assayed samples.

**Figure 6:**
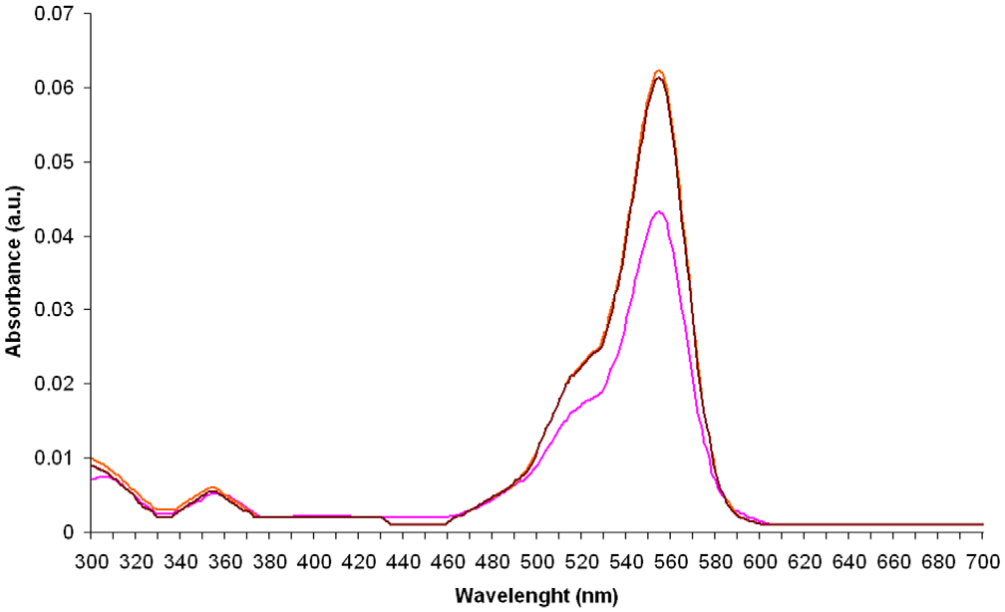
The UV-VIS absorption spectra of rhodamine B (brown and orange lines) in the presence of the different concentration of *E. coli* proteins, respectively, and the UV-VIS absorption spectrum of rhodamine B in the absence of *E. coli* proteins (pink line). The concentration of *E. coli* proteins in the first analyzed sample (brown line) was twice higher than the concentration of *E. coli* proteins (0.023 g L^−1^) in the second one (orange line), respectively. The concentration of RB (3.5×10^−4^ g L^−1^) was the same in the all assayed samples.

**Figure 7:**
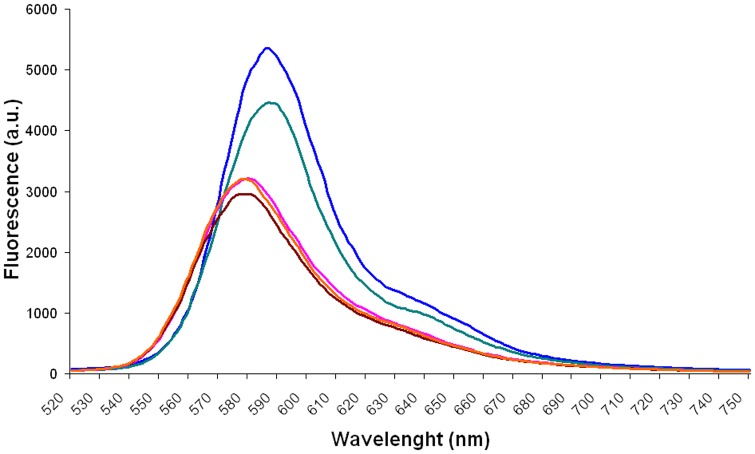
The emission spectra of rhodamine B (RB) in the presence of the different concentration of RSFP protein or *E. coli* proteins (Lys). The concentrations of RSFP protein (0.162 gL^−1^, green) and *E. coli* proteins (0.162 g L^−1^, orange) were twice higher than the level of RSFP protein (0.081 g L^−1^, blue) and *E. coli* proteins (0.081 g L^−1^, brown) in the analogous experiments, respectively. The concentration of RB was 1.225×10^−3^ g L^−1^.

The information about the final concentrations of RB, RSFP protein and *E. coli* proteins in the analyzed samples are given in the legends to the Figures 3,4,5,6,7, respectively.

### Crystallisation of RSFP protein

The 5 mL sample of RSFP protein was dialyzed from buffer A1 into 0.01 M Tris-HCl, pH 7.0. The resulting solution was concentrated on Viva-spin 10 kDa to a final protein concentration of 12 g L^−1^ and used for initial screening for crystallization conditions with a Hampton Index Screen. Initial crystals were obtained from 45% (w/v) PPG 400, 0.1 M Bis-Tris pH 6.5 and from other conditions containing MgCl_2_ and various PEGs. Combinations of polypropylene glycol 400 and magnesium chloride improved the quality of crystals. The optimization of the crystallisation conditions led to the following range of precipitants concentrations: 25–35% of PPG 400, 60–140 mM MgCl_2_ and the pH from 6.5–8.5 using Bis-Tris, HEPES and TRIS as a buffer. The best crystals were obtained with 35% (w/v) PPG 400, 75 mM MgCl_2_ and 0.1 M Bis-Tris pH 6.5. The crystals were grown using the hanging-drop vapour-diffusion technique at 293 K by mixing 1.5 µL of protein solution with 1.5 µL of the well mixture. Crystals of native RSFP protein appeared within 2 weeks. In order to obtain a complex of RSFP with RB, the native crystals were soaked overnight in the drop of well buffer enriched with 10 mM rhodamine B.

### X-ray diffraction data for *apo* RSFP and its complex with RB

Diffraction data for the *apo* RSFP at 1.98 Å and for its complex with rhodamine B at 1.90 Å resolution were collected using synchrotron radiation at BESSY Berlin, beamline BL14.2 on a MAR CCD 225 mm detector [12]. A single crystal of native RSFP (0.5×0.1×0.1 mm) was mounted in a nylon loop and flash-frozen to 100 K in a stream of N_2_ gas without any additional cryoprotection. The crystal of the RSFP/RB complex was washed in the well solution to remove excess of rhodamine B, and the diffraction experiment proceeded analogically as with the native crystal. Indexing, integration, and scaling of all diffraction images were performed in the *HKL2000* program [13]. The diffraction data of the RSFP/RB complex were reprocessed by *Mosflm* [14] and *Scala* [15], which gave better statistics.

### Site-directed mutagenesis

To confirm the crystallographic identification of amino acid residues involved in RB interactions with RSFP we performed a site-directed mutagenesis of *rsfp* gene. The triplets GAT, GAT, and AGC of the *rsfp* gene encoding D222, D224 and S22, respectively, were replaced with a GCT, GCT and GCC codon of alanine residue by using a QuikChange II XL Site-Directed Mutagenesis Kit (Agilent Technologies, USA), following the manufacturer's instructions. The primers containing the desired mutations (underlined): F_RSFP_D222A (CGTTAGCGCTAGTGACAGCTTTTGATT GTTGGCATCC) and R_RSFP_D222A (GGATGCCAACAATCAAAAGCTG TCACGCGCTAACG), F_RSFP_D224A (GCTAGTGACAGATTTTGCTTGTTG GCATCCAAATGAG) and R_RSFP_D224A (CTCATTTGGATGCCAACAAGCAAA ATCTGTCACTAGC), F_RSFP_S22A (CGCTATTATCGGCGGTGCCGGTCTG TATCAAATGC) and R_RSFP_S22A (GCATTTGATACAGACCGGCACCGCC GATAATAGCG), respectively, were designed based on the sequence of *rsfp* gene (GeneBank accession no. GQ202582.1). Plasmid pBADRSFP was used as the template to introduce the mutations into the *rsfp* gene and the resulting plasmids were sequenced. The selected recombinant plasmids with the proper variants of *rsfp* gene sequence were named pBADRSFP_D222A, pBADRSFP_D224A and pBADRSFP_S22A, respectively. Afterwards, the *E. coli* cells transformed with pBADRSFP-derivate plasmids were used for production of the new variants of RSFP protein following the procedure described above for wild type RSFP protein. Finally, the *E. coli*/pBADRSFP_D222A, *E. coli*/pBADRSFP_D224A, *E. coli*/pBADRSFP_S22A, *E. coli*/pBADMycHisA (negative control) and *E. coli*/pBADRSFP cell lysates were all examined with pink fluorescence assay.

## Results and Discussion

### Expression and purification of soluble RSFP protein

In our previous study [11], we demonstrated that the rhodamine B presence in the growth media and the expression of *rsfp* gene in *E. coli* cells is crucial for pink fluorescence of the *E. coli* colonies under UV illumination (λ = 312 nm). We have hypothesised that the interactions between the RSFP proteins and RB molecules could be the reason of the pink fluorescence phenomenon. To confirm this we constructed the set of pET22b(+)-derivate vectors for expression of the *rsfp* gene under T7 promoter control in the *E. coli* BL21(DE3)pLysS strain. Unfortunately, all variants of recombinant RSFP proteins were expressed as inclusions bodies. However, the fluorescence of RB excited by UV illumination in the presence or the absence of the refolded recombinant proteins wasn't different [11].

Therefore, in this study we examined the pBAD expression system (Invitrogen) for *rsfp* gene expression under araBAD promoter in the *E. coli* LMG 194 strain. Recently, this approach has been applied by us successfully in the expression of the cold-active β-d-galactosidase from *Paracoccus* sp. 32d [16]. In the case of this study, the pBAD expression system also allowed to achieve a soluble form of RSFP protein. The highest RSFP yield in the *E. coli* LMG194 cells was achieved by adding l-arabinose to the final concentration of 0.2% w/v, at A_600_ 0.5 and by further cultivation for 8 h at 30°C. RSFP was then purified by using the FPLC procedure. The SDS-PAGE of the purified RSFP protein revealed a protein band migrating near to 30 kDa corresponding to the expected molecular mass 32 kDa deduced from the RSFP protein sequence (GenBank accession no. ACS44285). The purification yield was about 25 mg of purified RSFP per litre of *E. coli* LMG194/pBADRSFP culture, as determined using the Bradford method [17]. Moreover, the RSFP was purified to ∼91% electrophoretical homogeneity according to the SDS-PAGE proteins profile densitometry analysis (Fig. 1).

The relative molecular mass of the native RSFP protein, determined by gel filtration, was ∼97 kDa suggesting that the examined protein is a trimer.

### The spectrophotometric studies of RB and RSFP

The pink fluorescence assay (PF assay) was firstly designed to compare the fluorescence of RB (sample A, Fig. 2) with the fluorescence of: (*i*) PBS buffer (negative control, sample B, Fig. 2), (*ii*) highly pure RSFP protein (sample D, Fig. 2), (*iii*) *E. coli* LMG194/pBADRSFP cell lysate (sample F, Fig. 2), and (*iv*) *E. coli* LMG194/pBADMycHisA cell lysate (sample H, Fig. 2), respectively. What was especially important for the PF assay was that all analyzed samples were illuminated with UV light (312 nm). The PF assay revealed the lack of any “pink” fluorescence for sample D containing highly pure RSFP protein. Moreover, the fluorescence intensity of this sample was comparable to the fluorescence intensities of the cell lysates of *E. coli* strains (samples F and H), respectively. However, under the assay conditions, only the sample A containing RB exhibited pink fluorescence. Therefore, we also decided to study the absorbance spectra (300–700 nm) and fluorescence spectra (520–750 nm, exc. 312 nm, bandwidth 9 nm) of RB and purified RSFP protein in PBS buffer. The absorbance spectrum of RSFP protein (Fig. 3, black line) revealed the highest absorbance in the range of wavelengths between 200 nm and 240 nm with maximum at 212 nm, which is indicative of the presence of peptide bonds in the RSFP protein. Moreover, the same spectrum revealed the presence of a second absorbance maximum at 280 nm, which indicates the presence of aromatic amino acids such as tryptophan residues in the RSFP protein [18]. What is important to note is that the absorbance of RSFP at 312 nm was much lower in comparison to the maximal absorbance of RSFP at 212 nm (Fig. 3, black line). Besides that, the absorbance of RB at 312 nm was significantly lower in comparison to its maximal absorbance at 554 nm (Fig. 3, pink line). However, at this experiment conditions the absorbance of RB was distinctly higher that the absorbance of RSFP at 312 nm.

Next, the emission spectrum of RSFP protein excited with 312 nm (Fig. 4, black line) revealed the low fluorescence intensities of RSFP in the range of visible light (400–750 nm). In comparison, the analogous emission spectrum of RB (Fig. 4, pink line) revealed significantly high peak fluorescence intensity in the range of 550–640 nm (green-yellow-red) with maximum at 578 nm. From these results, we concluded that the RSFP protein is not a new example of a “fluorescent” protein such as GFP protein characterized by the presence of the chromophore group composed of modified amino acid residues within the polypeptide chain [19].

Due to these results, we decided to expand and modify the pink fluorescence assay. In this new format, the PF assay was used to compare the fluorescence of pure RB solution (sample A, Fig. 2) with the fluorescence of RB in the presence of (*i*) highly pure RSFP protein (sample C, Fig. 2), (*ii*) *E. coli* LMG194/pBADRSFP cell lysate (samples E, Fig. 2), and (*iii*) *E. coli* LMG194/pBADMycHisA cell lysate (sample G, Fig. 2). From these results, we found that the presence of RSFP protein in the analyzed sample led to an increase of the fluorescence intensity of RB (samples C and E). Therefore, in the next stage of this study, we focus on the examination of the effect of the presence of RSFP protein in analyzed samples on the spectrophotometric properties of RB. In conclusion, we found that the whole absorption spectra of RB in the presence of RSFP protein were red shifted (Fig. 5, green and blue lines) in comparison to the absorption spectra of RB in the absence of this protein (Fig. 5, pink line) or the presence of *E. coli* proteins in analyzed samples (Fig. 6). Moreover, the wavelength of the maximum absorbance, λ_max_ = 554 nm, for RB in control samples containing *E. coli* proteins or pure RB in the PBS buffer, was the same as the wavelength of the maximum absorbance for RB in pure water [20]. On this basis, we found out that the residual amounts of *E. coli* proteins that are still present in the RSFP protein after purification process was not responsible for the observed red shifted absorbance spectra of RB in the presence of this protein. In addition, the analysis of the absorbance spectra of RB presented in Fig. 5 and Fig. 6, respectively, revealed the increase in the absorbance intensity of RB in the presence either RSFP protein or *E. coli* proteins under the experiment condition. Zhao et al. [21] proposed the hypothesis that the absorbance intensity change of RB in the polar solvent could be explained by solvent effects on solute-solvent interactions and the structure of RB molecules in solution and dispersions. In the polar solvent the RB molecule can exist in zwitterion form (RB±) or cation form (RB^+^), and the cations are capable of interactions with anions, for example chloride ions. In addition, the zwitterion form of RB and ion pair containing RB^+^ could form dimers [21], [22]. The dimers can be formed via electrostatic and van der Waals' forces. Hydrogen bonding is another major driving force for the formation of RB dimers in aqueous solution [21]. To sum up, based on the data presented by Zhao et al. [21] we suppose that the addition of RSFP protein or *E. coli* proteins to the aqueous solution of RB change the physicochemical properties of solvent that lead to disaggregation of RB dimers. Thus, the higher monomer concentration with high oscillatory strength could lead to the increase in the absorbance intensity of RB presented in Fig. 5 and Fig. 6, respectively.

Moreover, in order to collect more data for further conclusions, we achieved and analysed the emission spectra (exc. 312 nm, bandwidth 9 nm) of solution containing pure RB, RB in the presence of RSFP or RB in the presence of *E. coli* proteins. What was important to note, the fluorescence spectra of RB in the presence of RSFP protein (Fig. 7, blue and green lines), revealed: (*i*) the red shifts and (*ii*) the increase of the fluorescence intensities of RB emission maxima in comparison with the fluorescence intensities of pure RB or RB in the presence of *E. coli* proteins (Fig. 7, lines pink, orange and brown), respectively. In our opinion, in contrast to the presence of RSFP in the RB solution, the presence of *E. coli* proteins caused the quenching of the fluorescence of RB under the experiment condition.

On the other hand, interestingly, similar results of the pink fluorescence assay, the increase of fluorescence intensities, were observed only for samples containing RB in the presence of RSFP protein (samples C and E, Fig. 2).

In general, the red shifts in the RB spectra are diagnostic for transition of the RB molecule from the aqueous phase to the binding state and are the consequence of the change in the polarity and refractive index of the environment of the dye due to the adsorption process [20], [23], [24], [25], [26]. Taking this into account, we concluded that the presence of the red shifts of the RB spectra in the presence of the RSFP protein (Fig. 5 and 7) could be caused by the RB molecules adsorption on the surface of the RSFP protein. Therefore, to confirm the RB binding with the RSFP protein we decided to obtain the crystal structures of *apo* RSFP and its complex with rhodamine B. On the other hand, we strongly suppose that the observed increase in the fluorescence intensity of RB in the presence of RSFP (Fig. 7) could be caused by the change of the spectroscopic properties of dye molecules after binding with this protein.

### Crystal structures determination of *apo* RSFP and RSFP/RB complex

Analysis of the Matthews volume [27] for the native RSFP crystal indicates the presence of one molecule in the asymmetric unit (V_M_ = 2.37 Da·Å^−3^ solvent content 48.14%). The molecular replacement method was applied to solve the crystal structure of the enzyme in the *Phaser* programme [28] from the *CCP4* suite [29], using 5′-Deoxy-5′-methylthioadenosine phosphorylase from: *Aeropyrum pernix* (PDB: 1WTA), *Sulfolobus solfataricus* (PDB ID: 2A8Y) [30] and its homologue from *Sulfolobus tokodaii* (PDB ID: 1V4N) as models. The sequence identity of the investigated protein to the search models was 47%, 46% and 49% respectively. The last one had the best statistics in the *Phaser* program and this solution was used for further structure refinement. The structure was refined using *REFMAC* [31] and *PHENIX* [32] programmes. The crystal structure of RSFP/RB complex was solved by the rigid body refinement of the *apo* structure against complex diffraction data. The X-ray data collection and structure refinement statistics of the native crystal structure (PDB ID: 4GLF) and its complex with RB (PDB ID: 4GLJ) are presented in Table 1.

**Table 1:** X-ray data collection and crystal structure refinement statistics.

*Data collection*	*apo* RSFP (4GLF)	Complex RSFP/RB (4GLJ)
Radiation source	BL.14.2, BESSY, Berlin	BL.14.2, BESSY, Berlin
Wavelength [Å]	1.00	0.94
Temperature of measurements [K]	100	100
Space group	*P63*	*P63*
Cell parameters [Å]	*a* = b = 80.49, *c* = 81.34	*a* = b = 80.26, *c* = 81.26
Resolution range [Å]	50.0-1.98 (2.05-1.98)^1^	40.0-1.90 (2.00-1.90)^1^
Reflections collected	233379	160210
Unique reflections	20266	22360
Completeness [%]	96.6 (74.5)	95.4 (75.3)
Redundancy	11.52 (6.7)	7.2 (5.3)
<I>/<σI>	26.9 (2.8)	14.6 (4.0)
R_int_ 2	0.086 (0.378)	0.082 (0.342)
*Refinement*		
Reflections in the working/test sets	19185/1035	21376/936
R^3^/R_free_ [%]	15.3/20.9	15.5/20.3
Number of atoms (protein/solvent/ligands)	2230/191/0	2211/171/71
R.m.s. deviations from ideal		
bond lengths [Å]	0.020	0.018
bond angles [°]	1.89	2.02
<B> [Å^2^]	28.8	25.1
Residues in Ramachandran plot [%]		
most favoured regions	89.1	88.3
additionally allowed regions	10.9	11.7
generously allowed regions	0	0
disallowed regions	0	0

1 Values in parentheses correspond to the last resolution shell.

2 R_int_ = ∑_h_∑_j_ | I_hj_−<I_h_> |/∑_h_∑_j_ I_hj_, where I_hj_ is the intensity of observation j of reflection h.

3 R = ∑_h_ | | F_o_|−| F_c_| |/∑_h_ | F_o_| for all reflections, where F_o_ and F_c_ are observed and calculated structure factors, respectively.

R_free_ is calculated analogously for the test reflections, randomly selected and excluded from the refinement.

Both crystals, the *apo* RSFP and its complex with rhodamine B, belong to the hexagonal space group *P*6_3_.

### Architecture of the RSFP protein

**Figure 8:**
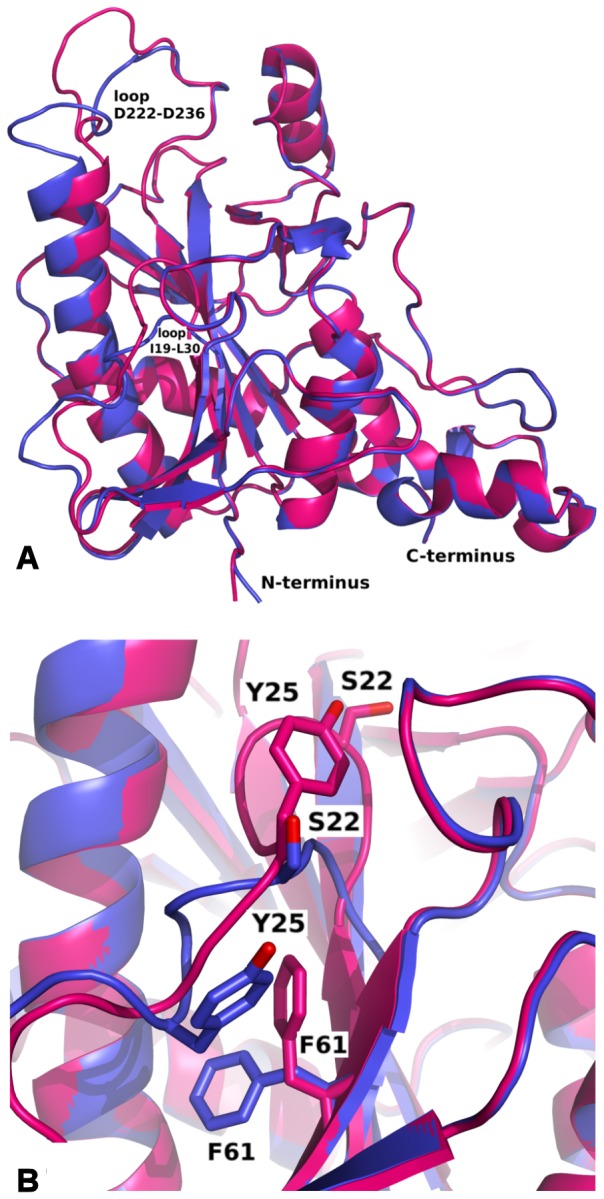
Alignment of *apo* RSFP form (blue) and its complex with RB (deep pink): (A) general view of monomers, (B) enlargement of loop I19-L30 with shown difference in location of S22, Y25 and F61 caused by interaction with phosphate ion and RB molecule.

**Figure 9:**
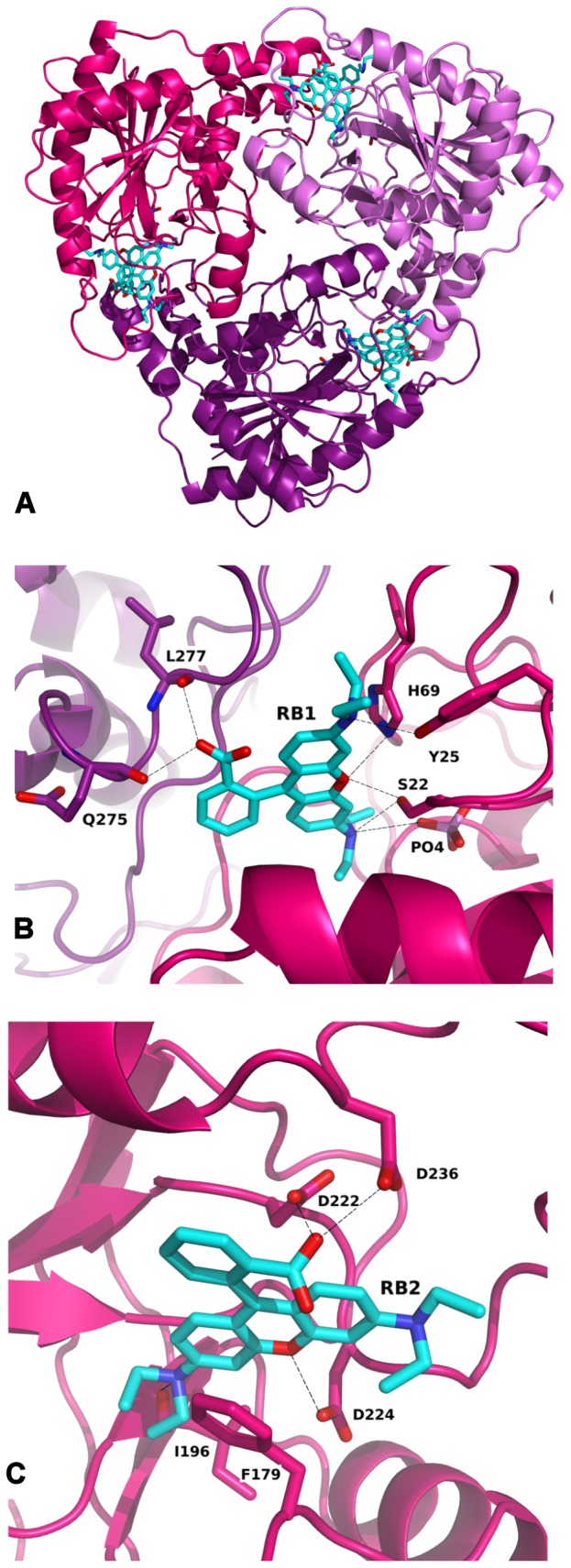
Interactions of RB with RSFP: (A) trimmer of RSFP with visible RB molecules on the interface between monomers; (B) binding site of RB1 interacting with neighbouring monomer (deep pink and violet); (C) binding site of RB2.

The monomer of RSFP has a mixed α-β architecture. The main structural motif is an eight stranded mixed β-sheet located in the centre of the monomer. On the one side of this sheet there are two long helixes, while on the opposite side there are five helixes with additional three stranded parallel β-sheets and a complicated network of loops (Fig. 8A). The native form of this protein is a trimer that is reflected in the crystal symmetry (Fig. 9A). In the presented crystal structures the monomer is an asymmetric unit. The trimer assembly is built on a crystallographic 6_3_-fold axis. Two structural elements: the M120 - R124 loop and the T181 – M191 α-helix are located close to the three-fold axis and these residues interact with equivalent residues from the symmetry related monomers. The longest helix 238A – 262S and the helix 268I – 276A located on the surface are responsible for the triangular shape of the trimer (Fig. 9A). Moreover, the structure of the native RSFP protein is similar to the homotrimer of human MTA-phosphorylase (PDB ID: 1CBO) [33] and MTA-phosphorylase from the hyperthermophilic archeon *Sulfolobus tokodaii* (PDB ID: 1V4N). However, the MTA-phosphorylase from the hyperthermophilic archeon *Sulfolobus sofataricus* (PDB ID: 1JDU) revealed a homohexameric structure, which can be described as a dimer of trimers [34].

On the other hand, the crystal structure of native RSFP didn't reveal the presence of any chromophore group assembled from amino acid residues present in RSFP protein. This result confirmed our earlier conclusion on this issue, presented in the discussion of the spectrophotometric studies of RSFP protein.

### Conformational changes of RSFP upon rhodamine B binding

The structures of the *apo* RSFP and its complex with RB are very similar; the rmsd of the superposed monomers is 0.571 Å. The main difference is observed in the RB binding site, as the flexible loop 222D – 236D in comparison to the *apo* structure changes conformation considerably (Fig. 8A). The biggest movement is visible for the part of the loop containing residues 230E – 236D. Also, the loop with residues 19I – 30L, located on the bottom of the RB binding pocket, changes its shape after rhodamine B binding (Fig. 8B). Two residues, 22S and 25Y, are shifted to the direction of the RB binding pocket. The movement of the 22S residue in comparison to the *apo* structure is 8.17 Å. The shift of this loop forces the conformational changes of the side chains of neighbouring residues. The most visible difference can be seen for 27M and 61F.

### Rhodamine B binding site in RSFP

**Figure 10:**
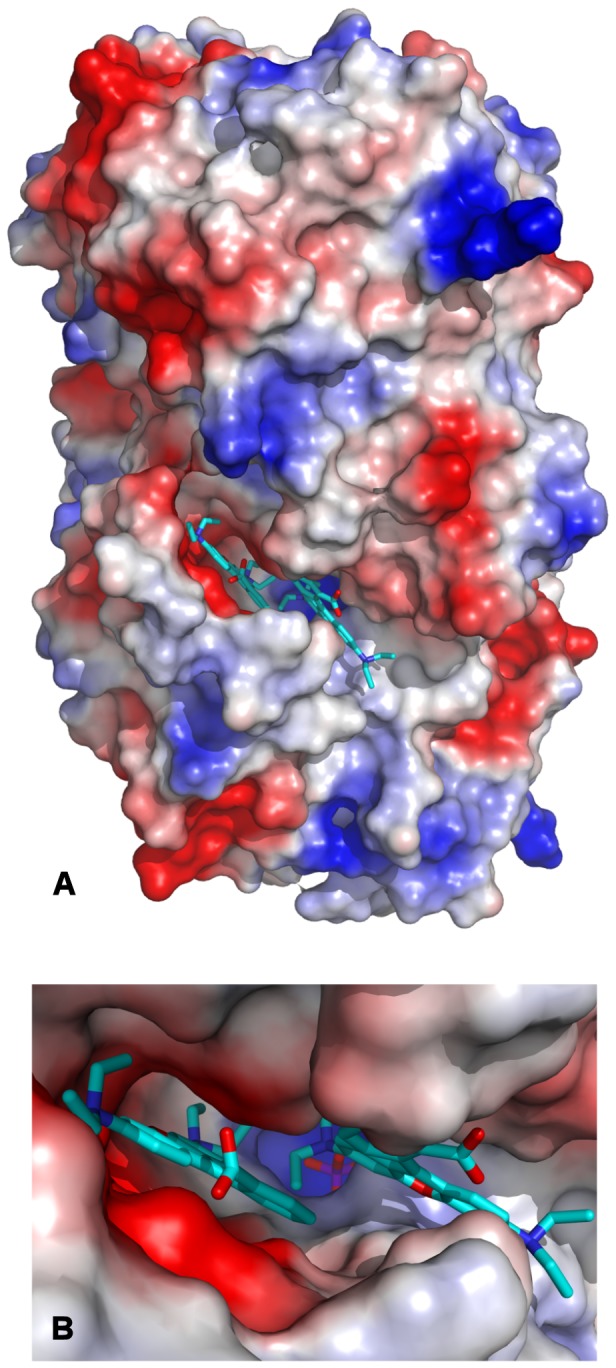
Surface electrostatic potential: (A) Trimmer of RSFP shown in orientation with the 3-fold axis in the figure plane; (B) zoom of the binding cleft.

The RB binding site is located in the cleft between neighbouring monomers creating a trimer (Fig. 9A, Fig. 10A and 10B). One wall of this cleft is created by a flexible loop 222D – 236D and the other by loop 177P – 182R and two loops from the neighbouring symmetry related monomers 134V – 140A and 268I – 279T. In the deepest part of the cleft there is a mobile loop 20G – 30L with the 22S residue on the top. This serine interaction with a phosphate ion causes change of this loop's conformation in comparison to the *apo* structure. The cleft is large enough to accommodate two RB molecules, which are labile and because of that, they were refined with a half occupancy. The RB1 molecule interacts with the phosphate ion, hydroxyl groups of 22S and 25Y, with the nitrogen of the imidazole ring from 69H and the main chain carbonyl oxygens of 275Q and 277L from the symmetry related monomer. Hydrophobic contacts are made with 243M, 242L, 239I and 278V from the symmetry related molecule (Fig. 9B). This binding place corresponds to the active pocket of MTA-phosphorylases and in case of the presence of methylthioadenosine molecules may be occupied by this substrate. Direct polar contacts of RB2 exist with the side chains of 222D, 224D, 236D, carbonyl oxygen of 196I and peptide nitrogen of 100G. Hydrophobic interactions by π-stacking take place between the ligand and 179F (Fig. 9C). Binding of both RB molecules into this pocket (Fig. 10A and 10B) required penetration to the crevice, likely associated with a movement of the flap loop.

### RSFP engineering

The presented results of the crystallographic studies revealed the presence of two possible RB molecule binding places in each RSFP monomer. To check, which of them is linked with the pink fluorescence phenomenon caused by the change of spectroscopic properties of the RB molecules binding to the RSFP protein, we decided to carry out site-directed mutagenesis in the *rsfp* gene. Based on the crystal structure of the RSFP/RB described in this work (PDB ID: 4GLJ) we chose to mutate residues S22, D222 and D224. Analysis of the hydrogen bonds created between RB and RSFP indicated that these amino acids could play the key role in the interactions of RB molecules with the RSFP protein. We found that the replacement of S22A revealed the lack of difference in the pink fluorescence between the recombinant colonies of *E. coli*/pBADRSFP_S22A and *E. coli*/pBADRSFP strains growing on LA medium supplemented with RB. In contrast, the replacement of D222A or D224A completely abolished the pink fluorescence of the recombinant colonies of *E. coli*/pBADRSFP_D222A or *E. coli*/pBADRSFP_D224A growing under the same growth medium and the same growth conditions as well. Moreover, the analogous results were found in the pink fluorescence experiment for the RSFP protein and its mutated variants. These experiments indicated that both the aspartic acid residues are crucial for binding of RB to the RSFP which causes the pink fluorescence phenomenon [11].

## Conclusions

First of all, in this paper we presented a successful method of production and purification of soluble RSFP protein expressed by employing the pBAD Expression System (Invitrogen). Secondly, we obtained the crystal structures of *apo* RSFP protein and its complex with rhodamine B. The analysis of the complex RSFP/RB crystal structure revealed that the RB binding site is located in the cleft between neighboured monomers creating functional trimer, and that this cleft is large enough to accommodate two RB molecules. Thirdly, the study of mutated variants of the RSFP protein allowed to link the pink fluorescence phenomenon of RB interactions with RSFP via residues D222 and/or D224. Fourthly, the present study excluded the possibility that the RSFP protein is a new fluorescent protein such as GFP characterised by the presence of an autocatalytically formed intrinsic chromophore. Finally, the presented results suggest that the fluorescence of recombinant *E. coli* colonies expressing RSFP have a reason in the changes of the spectroscopic properties of RB molecules by modifying its environment upon binding to the pocket between the RSFP monomers.

In summary, we have proved the existence of the connection between the RB molecules, the RSFP protein and the pink fluorescence phenomenon of the recombinant *E. coli* colonies expressing *rsfp* gene and growing on LB agar supplemented with rhodamine B reported in our previous study [11]. Moreover, to the best of our knowledge, this is the first study which revealed the specific interaction of RB molecules with protein and presented the crystal structure of RB/protein complex. What is important to note is that the studies of the toxicity of RB have suggested that the interaction between dye molecules and proteins are crucial for its toxic properties. Hence, recently, there is growing interest in studying of the interactions of RB molecules with proteins [35], [36]. Therefore, in the light of the presented data, we are going to examine the RSFP as methylthioadenosine phosphorylase (the enzyme of the methionine salvage pathway) and the effect of the RB binding on its enzymatic activity.
